# Editorial

**Published:** 1901-08-15

**Authors:** 


### EDITORIAL.

At the recent session of the National Association of Dental Faculties, a resolution was passed which makes it mandatory for all dental schools holding membership in that body to institute a four years' course of study with the term which begins in the fall of 1903. The length of the term will be for seven months, and the entrance requirements are to be raised to the equivalent of two years of high school work, this latter provision taking effect at the opening of the term which begins in the fall of 1902. This is an important step in dental education, and is significant of the progress being made not only by our educational institutions, but by the profession generally. We are, as a profession, sometimes inclined to assume all the credit for the great advances made in the profession, forgetting that the important if not essential factor in stimulating this advance is the demand of the public. And were it not for the fact that a prosperous and intelligent public is ever demanding a higher order of professional service, it is not likely there would be sufficient incentive to account for the ever-broadening variety, not to mention the high character of service which the up-to-date dentist is called upon to render. This extension of the college course is the natural result of this demand, as it is made necessary to adequately administer a considerably extended curriculum of studies. The colleges have not adopted this extension of the course from preference. In fact, it has forced its way against the wishes of the large majority of the schools in the association, as it means a larger equipment in the way of teachers and facilities, and for this reason may result in closing some schools which have insufficient financial resources. It is also expected that it may decrease the number of students entering the schools.and thus work a hardship to the weaker schools, and the poor boys. However this may be, if it shall result in a better educated class of dentists, even at a loss of numbers, the people will receive the benefit. Personally we feel that there will be drawn into the profession a more intellectual class of young men, their training will consequently become easier and the results will be so much more satisfactory that an adequate compensation for the numerical loss will be had.

---

ANOTHER expression of the sentiment that the educational advantages and requirements of such as seek to enter the profession through the dental colleges should be of a high order and as nearly uniform in all parts of the country as may be practicable is manifest in the action taken to compel all colleges in the Faculties Association to conduct their exercises during the part of the day usually devoted to educational exercises. This action discredits all so-called "sundown" or night schools, and was taken for the reason that their students are not physically and mentally in the best condition to receive instruction, because they have spent their best energies at other secular callings during the part of the day in which one is normally most effective. It would seem that if there should prove to be a justifiable demand for such institutions, their terms should be correspondingly extended and their facilities placed on a par with those of the best graded schools.

THE President's address of the National Dental Association this year calls attention again to the fact that under the present constitution of that body it is impracticable to secure a program for it, or to administer its affairs in such a way as to do justice to such as desire to contribute to the program or take part in its proceedings. The organization has grown so large and so many papers are offered that there is no adequate time allowed for their consideration. The address recommends a revision of the constitution which will divide the organization into three sections, before which papers appropriate to each shall be read simultaneously, somewhat after the plan of the American Medical Association, and that general meetings shall be held at proper intervals during the session for the consideration of executive affairs only. It is well known that some of the best writers and workers in our profession have declined to present papers to this body for the reason that no adequate consideration can be secured, preferring to present their work in smaller societies where time will be given and competent persons will be present to discuss them. This fact has brought into the program of the National Association, where only the very choicest productions of the profession ought to appear, hurriedly written or poorly considered essays, which have discredited the work of this organization to such an extent that it has come to be of no great credit or honor to be placed on its program. We trust that this matter may be remedied as it should be, for the credit and honor of the profession. The program is the vital element of this organization, and it should be the first aim and purpose to make it such as will command the respect and interest of the dental world. This can only be accomplished by some well-executed plan of censorship. It is not possible to present in a program all that is offered, even if it should seem expedient. It ought to be practicable by some competitive scheme to secure sufficient high grade papers to make up a program that will command attention. If this is not practicable then let us have a program committee, who shall design a program for each session and secure suitable persons to carry it into effect. This method is carried out in other societies with the very best results, and conditions in the National Association are not so materially different as to make it impracticable.

---

Dr. Otto Arnold has resigned irom the faculty, and as dean, of the Dental Department of the Ohio Medical University and Dr. L. P. Bethel will succeed him. Dr. Arnold's health is such that it is inexpedient for him to continue this work. The department has prospered under his administration, and it is hoped that by a rest and change of occupation, for a time, that his health may be materially restored. Dr. Bethel will move to Columbus in the fall and take charge of the school. He will still continue his work as editor of the *Ohio Journal*, but will resign as a member of the Ohio State Board of Dental Examiners. We most cordially commend Dr. Bethel to the faculty and students of the Ohio Medical University, and trust that they will support him loyally in his efforts to make their school what it should be, and what it has many reasons for being, one of the strongest and best schools in the West.

---

A rather dramatic incident occurred at the recent meeting of the National Association of Faculties when the sheriff of Milwaukee stepped into the meeting and served papers on the officers enjoining the Association from expelling the Dental Department of the National University of Washington, D. C., from membership, because it did not conform to the Association's ideals as to equipment and teaching. Vice-President Litch, who was in the chair, was equal to the occasion however.

